# A General Overview on Non-coding RNA-Based Diagnostic and Therapeutic Approaches for Liver Diseases

**DOI:** 10.3389/fphar.2018.00805

**Published:** 2018-08-15

**Authors:** Sanchari Roy, Christian Trautwein, Tom Luedde, Christoph Roderburg

**Affiliations:** Department of Medicine III, University Hospital RWTH Aachen, Aachen, Germany

**Keywords:** ncRNA, miRNA, lncRNA, liver, HCC, therapy

## Abstract

Liver diseases contribute to the global mortality and morbidity and still represent a major health problem leading to the death of people worldwide. Although there are several treatment options available for Hepatitis C infections, for most liver disease the pharmacological options are still limited. Therefore, the development of new targets against liver diseases is of high interest. Non-coding RNA (ncRNA) such as microRNA (miRNA) or long ncRNA (lncRNA) have been shown to be deeply involved in the pathophysiology of almost all acute and chronic liver diseases. The emerging evidence showed the potential therapeutic use of miRNA associated with different steps of hepatic pathophysiology. In the present review, we summarize emerging insights of ncRNA in liver diseases. We also highlight example of ncRNAs participating in the pathogenesis of different forms of liver disease and how they can be used as potential therapeutic targets for novel treatment paradigms. Furthermore, we describe an overview of up-to-date clinical trials and discuss about its future in clinical applications. Finally, we highlight the role of circulating ncRNAs in diagnosis of liver diseases and discuss the challenges and drawbacks of the usage of ncRNAs in clinical setting.

## Non-Coding RNA

Studies on the transcription landscape of the human genome show that only 2% of the genome encodes for proteins, while 98% of the genome consists of non-coding sequences. Just recently, it has become clear that the non-protein coding parts of the genome cannot be simply considered as ‘junk’ RNA but have important functions e.g., in the regulation of gene expression (Pertea, 2012). Non-coding RNA (ncRNA) bind to both DNA and RNA in a very specific way, potentially leading to their degradation or alterations in their transcription, processing, editing and translation (Geisler and Coller, 2013). In addition, ncRNAs generate a complex regulatory network by competing among each other for binding to mRNAs, behaving as competing endogenous RNA (ceRNA) (Ragusa et al., 2017;Yamamura et al., 2018). In this context, the modified version of Francis Crick’s ‘central dogma’ illustrates the role of ncRNA, where the information passed from DNA to RNA can either be directly transformed into protein or as regulatory RNA that influences the transcription or translation of other coding or non-coding genes (Peschansky and Wahlestedt, 2014; Cobb, 2017).

Non-coding RNA have become subject of translational research due to its involvement in the pathophysiology of a broad range of human diseases including genetic disorders, inflammatory diseases and various cancers. The unique nature of RNA, which can be synthesized energetically and rapidly, makes them a promising target for drug development. Along with the classical functional subtypes such as ribosomal RNA (rRNA), small nuclear RNA, small nucleolar RNA and tRNA, ncRNA are divided into two major classes based on the transcript length. Small ncRNA (≤200 nucleotides) includes microRNA (miRNA), small interfering RNA (siRNA) and PIWI- interacting RNA, while RNAs >200 nucleotides in length are summarized as long ncRNAs (lncRNAs) (Gomes et al., 2013; Adams et al., 2017). In comparison to miRNAs, lncRNA lack open reading frames and are mostly tissue specific (Derrien et al., 2012; Anastasiadou et al., 2018). In this review, we discuss the role of ncRNA in liver diseases. We highlight important examples of ncRNA participating in the pathogenesis of different forms of liver disease and how they can be used as therapeutic tools/ targets for novel treatment paradigms. Furthermore, we attempt to give an overview of up-to-date clinical trials featuring ncRNA.

## microRNA

microRNA are small ncRNA molecules (18–24 bp in length) that negatively regulate the expression of their target mRNAs and lead to either their translational suppression, cleavage or decay. More than 2500 miRNA are annotated in human according to miRBase version 21. miRNAs are predicted to target more than 60% of protein-coding genes, exerting a pleiotropic effect on biological networks (Jansson and Lund, 2012). Binding of miRNA to the 3’UTR of specific mRNA targets occur through a specific miRNA region called ‘seed region’ consisting of a contiguous string of at least 6 nucleotides at position 2 beginning of 5′ of the molecule (Thomson et al., 2011). The molecular mechanism concerning block of translation or mRNA degradation is mediated by an RNA-induced silencing complex (RISC) that includes Argonaute (AGO) protein family (Valencia-Sanchez et al., 2006). miRNA control several cellular processes including proliferation, differentiation, cell-death, metastasis and angiogenesis that contributes to the pathogenesis of liver diseases (Ha, 2011).

## The Role of miRNA in the Liver

Since their discovery in 1993, tremendous efforts were made to clarify the physiological role of miRNA in tissue development, homeostasis and regeneration of the liver (Schueller et al., 2018). miR-122 represents the most abundant hepatic miRNA, contributing about 70% of all hepatic miRNA (Chang et al., 2004; Girard et al., 2008). Inhibition of miR-122 was associated with decreased plasma concentrations of different lipids (such as cholesterol) and increased hepatic fatty-acid oxidation, resulting in a reduction of hepatic fatty-acid and cholesterol synthesis (Esau et al., 2006). Furthermore, miR-122 inhibition was linked to the development of hemochromatosis by up-regulating the expression of human hemochromatosis protein (Hfe), hemojuvelin (Hjv), bone morphogenetic protein receptor type 1A (Bmpr1a) and the hepcidin antimicrobial peptide (Hamp) (Castoldi et al., 2011). Along with miR-122, a broad variety of other miRNA have been shown to be deeply involved in the physiology and pathophysiology of the liver. Interesting insights on the role of miRNA in liver development were provided by Hand et al., illustrating significant downregulation of miR-122, miR-192, and miR-194 in conditional knockout of DICER1 in hepatoblast-derived cells. Dicer1^flox/flox^ mutants displayed no change in phenotype directly after birth, but the progression of hepatocyte damage at 2–4 months of age was observed, as demonstrated by increased aspartate aminotransferase (AST) and alanine aminotransferase (ALT) levels. Moreover livers of these mice were enlarged most likely due to an increased hepatocyte proliferation and apoptosis (Hand et al., 2009). A comprehensive review on miRNA function in liver development is given in (Chen and Verfaillie, 2014), moreover important ncRNAs in liver diseases are cell-specifically given in **Table 1**. In conclusion, miRNAs demonstrate their function in maintaining liver homeostasis, and their involvement in acute and chronic liver diseases.

**Table 1:** ncRNA altered in liver diseases.

	Targets	Reference
**Cholangiocyte specific ncRNA**		
miR-124	STAT3, IL-6R	Xiao et al., 2015
H19	SHP	Li et al., 2018
**HSC-specific ncRNA**		
miR-29	IGF-I, PDGF-C, HSP47, Collagens	Kwiecinski et al., 2011; Roderburg et al., 2011
miR-30	KLF11	Tu et al., 2015
miR-200	α-SMA, β-catenin, TGFβ-2	Sun et al., 2014
miR-122	P4HA1, FN1, SRF	Li et al., 2013; Zeng et al., 2015
miR-21	PTEN, API, SPRY2, HNF4, PDCD4	Wei et al., 2013; Zhang et al., 2013
miR-34a	ACSL1	Yan et al., 2015
MEG3	Iκbα	Chen et al., 2016
MALAT1	CXCL5	Leti et al., 2017
NEAT1	miR-122, KLF-6	Yu F. et al., 2017
PVT1	miR-152	Zheng et al., 2016
GAS5	miR-222	Yu et al., 2015
lincRNA-p21	p21	Zheng et al., 2015
**Hepatocyte-specific ncRNA**		
miR-122	CyclinGl, ADAM10, IGF1R, SRF	Bai et al., 2009; Hou et al., 2013
miR-192	Zebl, Zeb2	Kim et al., 2011; Roy et al., 2016
H19		Goyal et al., 2017
Hand2	C-met	Wang et al., 2017
00321	CyclinBl	Wu et al., 2018
ARSR	Akt/SREBP-lc	Zhang et al., 2018
HULC	miR-186	Wang et al., 2018
**Kupffer cells-specific miRNA**		
miR-155	Smad3, C/EBPβ	Csak et al., 2015
miR-223	Caspase 3	Yang et al., 2014
HIF1A-AS		Wu et al., 2018


## Therapeutic Use of miRNA in Metabolic Liver Diseases

Obesity and type 2- diabetes mellitus represent epidemic metabolic disorders affecting millions of people worldwide due to different genetic backgrounds, lifestyles and environmental stimuli. In the last years, it was demonstrated that miRNA represent key regulators of metabolism. While miR-33a/b and miR-122 directly controls cholesterol and lipid metabolism in concert with their host genes (reviewed in Moore et al., 2011; Fernandez-Hernando et al., 2013; Singh et al., 2017; Aryal et al., 2017), the SREBP transcription factors and represent the best studied miRNA in the context of metabolic liver diseases and NASH, miR-103 and miR-107 have recently emerged as new regulators of insulin and glucose homeostasis in the context of metabolic liver diseases (Joven et al., 2012; Chakraborty et al., 2014; Vienberg et al., 2017). miR-103 and miR-107 are located in introns in the pantothenate kinase 1-3 (PANK1-3) genes, are up-regulated in livers of leptin-deficient (ob/ob) mice (Xie et al., 2009). Antisense-mediated silencing of miR-103 and miR-107 improved insulin sensitivity and glucose homeostasis (Trajkovski et al., 2011) whereas overexpression (predominantly in adipose tissue) was sufficient to cause defects in glucose homeostasis in these mouse models (Naar, 2011; Rottiers and Naar, 2012) Based on these results, Regulus^TM^ developed a GalNAc-conjugated anti-miR-103/107 (RG-125), that was tested for the treatment of nonalcoholic steatohepatitis (NASH) patients who suffered from type 2 diabetes in parallel. (Lovis et al., 2008; Zhao et al., 2009). In the third quarter of 2016, Astrazeneca launched a Phase I/IIa study involving subjects with type II diabetes and nonalcoholic fatty liver disease (NAFLD). The study is anticipated to be complete in December 2017 and in case of positive results might represent a new milestone in translating miRNA into clinical routine.

## Therapeutic Use of miRNA in Non-Metabolic Liver Diseases

Liver fibrosis and cirrhosis represent the common end-point of chronic liver injury. Hepatic stellate cells (HSC) represent the main cell type involved in the development of liver fibrosis as, upon inflammatory stimuli they transform into a myofibroblast-like phenotype and start to secrete extracellular matrix proteins such including different collagens, ultimately leading to hepatic fibrosis. Various authors have demonstrated that the expression of large number of miRNAs is altered during the process of HSC activation (Roderburg et al., 2011 Hepatology). While e.g., miR-29c^∗^, miR-501, miR-349, miR-325-5p, miR-328, miR-143, and miR-193 displayed a significant upregulation, miR-341, miR-20b-3p, miR-15b, miR-16, miR-375, miR-122, miR-146a, miR-92b, and miR-126 were found to be downregulated (Guo et al., 2009a,b; Maubach et al., 2011; Lakner et al., 2012). Just recently it was demonstrated that miR-29a transgenic mice were less prone to develop liver fibrosis after undergoing bile duct ligation surgery compared to wild-type mice. Moreover, the group of Knabel and colleagues demonstrated that the systemic delivery of scAAV8-encoded miR-29a ameliorates liver fibrosis in CCl4 treated mice (Knabel et al., 2015; Yang et al., 2017). All together, these data provide a strong evidence for a potential therapeutic use of miRNA and especially of miR-29 (summarized in (Deng et al., 2017)) in the management of patients with liver fibrosis.

Hepatocellular carcinoma (HCC) is the most important primary liver tumor representing the third leading cause of all cancer related deaths worldwide. Its curative treatment encompasses liver transplantation, liver resection and radiofrequency ablation (RFA) when the tumor is localized within the liver (Petrowsky and Busuttil, 2008). Unfortunately, in most cases HCC is diagnosed in later disease stages, when treatment options are limited and curation cannot be achieved. In these cases, Sorafenib (Nexavar) is the only approved substance for systemic therapy (Raza and Sood, 2014; Finn et al., 2018). However, the efficacy of Sorafenib is only moderate with a median survival of 10.7 months in the SHARP trial, which compared Sorafenib against placebo in patients with advanced (BCLC C) HCC (Rimassa and Santoro, 2009). Thus, in patients, that are not eligible for curative treatment approaches the overall survival is still poor and novel therapeutic targets are urgently needed.

The advantages of miRNA-mediated therapy over small molecule drugs (such as Sorafenib) include (i) the ease of chemical modification of oligonucleotides (Lennox and Behlke, 2011), (ii) metabolic ability through nucleases and (iii) multiple gene targeting ability to modulate cellular and biological processes (Garzon et al., 2010). Inhibition and restoration of oncogenic and tumor-suppressive miRNA can be therapeutically manipulated through different technologies for example antisense oligonucleotides (ASO) or miRNA sponge and synthetic miRNA mimics. As competitive inhibitor of miRNA, ASO known as anti-miRNA or antagomir anneals to the mature guide strand and induces degradation of duplex formation. Krutzfeldt et al. (2005) administered intravenous chemically modified miR-122 with cholesterol or with 2′-*O*-methyl linkage and phosphorothioate *in vivo* of which the former modification increases cellular uptake in hepatocytes, whereas the latter improve the binding affinity and prevent degradation by nuclease, respectively. Although they can be delivered intravenously, they have poor stability. In comparison to ASO, locked nucleic acid anti-miRs (LNA-anti-miRs) are stable and specific due to the presence of phosphorothioate in their backbone. An effective anti-miRNA therapy, Miravirsen is a 15-nucleotide LNA anti-miR against mature miR-122, known to promote HCV RNA accumulation within the cells (Lanford et al., 2010; Janssen et al., 2013). This anti-miR was tested on 36 patients with chronic HCV genotype I infection within a phase II clinical trial (NCT01200420, funded by Santaris Pharma). It was found that subcutaneous injection of Miravirsen resulted in prolonged decrease in HCV RNA in a dose- dependent manner. In addition, the safety of Miravirsen in patients was evaluated and no liver-related complications were found. Just recently, van der Ree et al. (2017) presented results from a phase 1B study that assessed safety and antiviral efficacy of a single dose of RG-101, a hepatocyte targeted *N*-acetylgalactosamine conjugated oligonucleotide that antagonizes miR-122, in patients with chronic HCV infection with various genotypes (van der Ree et al., 2017). 32 patients were enrolled of which 28 patients received RG-101. Similar to Miravirsen, treatment with RG-101 reduced viral in all treated patients within 4 weeks, and lead to a sustained virological response in three patients. Considering the positive safety and efficacy profile of both Miravirsen and RG-101 it seems likely that antagonizing miR-122 might will enter clinical application within the next years in patients with hepatitis C virus infection. Notably, phase 2 studies analyzing are the efficacy of a combination of RG-101 with direct-acting antivirals are underway (EudraCT 2015-001535-21). Of note treatment with Miravirsen and RG-101 were associated with a prolonged but reversible decrease in total plasma cholesterol levels, which is in line to their predicted targets. Obviously, these data might be of potential impact on the treatment of metabolic liver diseases such as NAFLD or NASH (Szabo and Csak, 2016). However, until now no data from clinical trials are available to support a use of Miravirsen or RG-101in these patients.

miRNA sponges contain multiple binding sites complementary to seed sequence (nucleotides 2-7) of the miRNA of interest (Ebert et al., 2007), therefore the miRNA family having same seed sequence can be targeted. Up-regulation of miR-221 in HCC was associated with tumor stage, metastasis and recurrence period after surgery (Gramantieri et al., 2009). An infection of Hep3B cells with adenovirus vectors encoding ‘miR-221 sponge’ reduces cell viability and induces apoptosis (Moshiri et al., 2014). A very recent publication by He et al showed miRNA sponge against miR-351 alleviated hepatic fibrosis by targeting the vitamin D receptor (He et al., 2018).

microRNA mimics are double-stranded synthetic miRNA molecules which, when administered in cells, are processed into a single-stranded antisense (guide-) strand identical to the miRNA-strand of interest, while the less stable (passenger-)strand can be attached to cholesterol in order to increase the cellular uptake (DiMarco and Fernandez, 2015). Other than mimics, loss of tumor-suppressor miRNA can also be restored by adenovirus-associated vectors with cloned miRNA of interest (Liu and Berkhout, 2011). TP53 is a multifunctional transcription factor that controls cell cycle progression, programmed cell death and DNA replication and repair (Stegh, 2012). It is frequently mutated therefore behaves as tumor suppressor in human cancers. Loss of miR-34a in HCC is linked to the status of p53. A small molecular modulator, Rubone has been shown to increase the occupancy of p53 on the miR-34 promoter that induces the expression of miR-34 (Xiao et al., 2014), leading to anti-tumor effect in xenograft HCC mice. MRX34 is a therapeutic- based liposomal miR-34 mimic that has been undergoing phase I clinical trial (NCT01829971) in patients with advanced solid HCC tumors (Beg et al., 2017). In a subset of 14 patients with refractory advanced tumors, application of MRX34 intravenously twice weekly has shown evidence of antitumor activity. However, due to severe immune related adverse events the trial was halted and a clinical trial for MRX34 specifically in patients with malignant melanoma could not been started.

## Circulating miRNA

The sequential progression from fibrosis to cirrhosis results in dysplastic nodules, finally culminating into HCC. Several research groups have analyzed single miRNAs or miRNA panel with a diagnostic potential for liver fibrosis in patients with chronic liver diseases (summarized e.g., in Roy et al., 2015; Loosen et al., 2017; do Amaral et al., 2018). Notably, for some of these miRNAs (e.g., miR-22, miR-34, and miR-122), it could be demonstrated that their diagnostic value of is superior to that of classical markers including alanine aminotransferase, CK-18, FIB-4 and APRI, highlighting the potential of miRNAs in the context of liver diseases (Anadol et al., 2015; Appourchaux et al., 2016; Liu et al., 2016). Besides liver fibrosis, circulating miRNA has been considered as potential non-invasive biomarkers detected in plasma and serum that not only stratifies the patients with early-stage tumors, but also the patients with advanced stage tumors, tumor recurrence and chemosensitivity. Several studies have shown the importance of cell-free circulating miRNA to distinguish between different stages of HCC with respect to control donors. In a large study, Zhou et al. (2011) analyzed alterations in circulating miRNAs in plasma from 934 patients [chronic hepatitis B (CHB), cirrhosis and HBV-related HCC] from three different independent cohorts. In these analyses, a panel of seven miRNA including miR-122, miR-192, miR-21, miR-223, miR-26a, miR-27a, and miR-801 that provided the possibility to differentiate HCC patients from healthy controls and even from patients with liver cirrhosis was identified. Yet another intriguing finding on circulating miRNA has been shown by Chen et al. (2013) in which an optimal plasma miRNA signature was established to diagnose patients with early stage liver cirrhosis. By the application of genome-wide microarray, they identified miR-106 and miR-181b to be down-regulated and up-regulated respectively, in independent training and validation cohorts comprising of 128 CHB-related cirrhosis patients, 79 CHB patients and 137 healthy donors. Furthermore, the diagnostic performance of the miRNA classifier in CHB-related silent cirrhosis was improved and higher than the Area under curve (AUC) of single clinical parameter, including total bilirubin, AST, ALT and prothrombin time.

Lin et al. (2015) recently attempted to identify serum miRNA combinations that could not only detect the presence of clinical but also predict the development of HCC in at-risk patients. This three-stage study from China involved healthy controls, inactive HBsAg carriers, individuals with CHB, individuals with hepatitis B-induced liver cirrhosis, and patients with diagnosed HCC. The study demonstrated that a miRNA classifier (*Cmi*) containing seven differentially expressed miRNAs (miR-29a, miR-29c, miR-133a, miR-143, miR-145, miR-192, and miR-505) was suitable for diagnosis of HCC. The value of Cmi for the detection of preclinical HCC was higher than that of AFP. In detail the sensitivity of *Cmi* was 29.6% 12 months before clinical diagnosis, 48.1% 9 months before clinical diagnosis, 48.1% 6 months before clinical diagnosis, and 55.6% 3 months before clinical diagnosis (Lin et al., 2015), highlighting the potential of circulating miRNA in the context of HCC diagnosis and surveillance of patients at high risk for developing HCC.

## lncRNA in Liver Disease

The first functional lncRNA was introduced 20 years ago. Functionally it was shown that this lncRNA is responsible for X-chromosome inactivation and lacks an ORF. The low detection limit of lncRNA is due to the tissue-specific expression and low conservation of lncRNA within the different species. The most widely described class of lncRNA is natural antisense transcript (NAT) in *cis* position.

## HCC, Liver Fibrosis and Metabolic Disease

The aberrant expression of lncRNA in HCC plays a role in the modulation of malignant phenotypes. Discovered in 2007, HULC (highly up-regulated in liver cancer) is <500 nt highly conserved lncRNA and is regarded as the most up-regulated gene in HCC (Panzitt et al., 2007). Elevated levels of HULC were observed in liver cancer or cancer metastasizing to the liver such as colorectal cancer with hepatic metastasis (Yu X. et al., 2017). Several *in vitro* studies have been performed to understand the regulation of HULC in cellular pathways. HULC is a part of regulatory network acting as a ‘sponge’ that down-regulates few miRNA, including miR-372, miR-9 and miR-107 which in turn activate the transcriptional factors CREB, PPARA and E2F1, respectively. This network ultimately results in tumorigenesis-related gene reprogramming, including proliferation and angiogenesis. Expression of HULC was strongly associated with HBV X (HBx) protein, an oncogenic viral protein. HBx up-regulates HULC levels which in turn promotes hepatoma cell proliferation by suppressing p18 (Du et al., 2012). To further evaluate the therapeutic role of HULC in liver cancer *in vivo*, the acceleration and decrease in tumor volume was observed in nude mice transplanted with HULC overexpressing or siRNA-HULC transfected hepatoma cells.

H19, a maternal expressed and paternal 2.7 kb gene is located near the telomere region of chromosome 11. Its abundant expression in embryogenesis contains miR-675on 1st exon, whose excision is mediated by HuR, stress response RNA binding protein. High expression of H19 is associated with advanced HCC stages and prognostic significance correlates with the predictive value of tumor recurrence (Raveh et al., 2015). H19 is linked to HCC tumor with inflammatory conditions including viral hepatitis, alcoholic and NASH. The oncogenic potential of H19 was confirmed by reduction in tumor volume caused by siRNA-H19 transfected Hep3b cells. miR-675 encoded H19 controls cellular growth via suppression of E-cadherin (Matouk et al., 2014).

Other lncRNA - MALAT-1 (metastasis-associated lung adenocarcinoma transcript) is ubiquitously expressed in liver, lung, kidney, spleen, heart and testis. High expression of MALAT-1 in the liver was at high risk of cancer recurrence after surgery. Reduction of MALAT-1 in HepG2 cells reduced tumor progression, cell proliferation and viability. The function of MALAT-1 has been studied in liver fibrosis where down-regulation of MALAT-1 in HSC decreased the expression of myofibroblast markers and restored the level of SIRT1 (Lai et al., 2012; Wu et al., 2015; Hou et al., 2017).

In addition to oncogenes, lncRNA act as tumor suppressors such as Meg3, AOC4P, INXS and Dreh. Meg3 is predicted to be an independent prognostic factor for HCC patients due to positive correlation of its low expression to worse survival and relapse-free survival. Overexpression of MEG3 induces apoptosis and decreases anchorage-dependent and –independent cell proliferation in both, HCC and liver fibrosis models (Braconi et al., 2011; He et al., 2014). The direct therapeutic effect of MEG3 on tumor growth in liver cancer and fibrotic liver has not been yet established. Similarly, amine oxidase, copper containing 4 pseudogene (AOC4P) correlated with poor prognostic outcomes in 108 HCC patients and reduced cell growth, migration and promoted EMT *in vitro*. Therapeutic application of AOC4P overexpressing SK-Hep1 cells into nude mice has been shown to significant reduced tumor growth and lung metastasis 8 weeks after tail vein injection (Wang et al., 2015).

To understand the role of lncRNA in liver metabolism, one study has reported an inter-genic liver-enriched lncRNA LncLSTR whose deletion in mice reduces plasma triglyceride (TG). LncLSTR has an impact on FXR/apoC2 pathway that modulates several metabolic pathways including bile acid synthesis and lipid homeostasis. However, more studies has to be performed to understand the full potential as a therapy (Li et al., 2015). High-throughput expression profiling of lncRNA in 48 NASH patients has identified liver-specific high expression of RP11-484N16.1 with positive correlation of NASH grade and NAFLD score playing a role in cell growth (Atanasovska et al., 2017).

## Circulating lncRNA

An increasing number of circulating lncRNA are reported as potential disease biomarkers. However, the need of highly sensitive detection of lncRNA in circulation is required for their low expression to understand diagnosis, prognosis and prediction of recurrence. For example, in addition to tissues, HULC was found to be up-regulated in the plasma of HCC, colorectal patients metastasized to the liver and in hepatitis B positive patients, indicating circulating HULC as diagnostic biomarker. Yet another lncRNA-AF085935 distinguishes both HCC patients from healthy individuals as well as HCC patients from hepatitis B-infected patients (Lu et al., 2015).

## Challenges in Therapeutic Targeting of ncRNAs in Liver Disease

microRNA-targeting therapeutics could be a powerful strategy for cancer treatment. Although there are number of reported successful preclinical studies regarding miRNA therapeutics, only a few of them have proceeded into clinical trials. The challenges for the development of miRNA-based therapeutics include off-target effect and ineffective delivery to the site of interest. Insufficient delivery of synthetic unmodified ‘naked’ oligonucleotides to a specific target to achieve maximal target inhibition is caused by the delivery obstacles such as degradation by serum and cellular nucleases or their poor uptake due to size and negative charge. The most important aspects of overcoming these obstacles are chemical modification with a proper sequence and optimal concentration of oligonucleotides. The prevention of the passage of hydrophilic negatively charged oligonucleotides through negatively charged plasma membranes should also be taken into account (Burnett and Rossi, 2012; Winkler, 2013; Juliano, 2016). Moreover the efficacy of ncRNA delivery is tissue dependent: liver, kidney and spleen are more accessible than other tissues in terms of sufficient delivery, although the side effects such as autoimmunity should be considered when designing oligonucleotides for cancer treatment. Off target effects result in silencing of genes other than the target gene along with potential immune response and toxicity due to chemical modification. miRNA mimics, administered therapeutically are detected by the innate immune system TLR, which in turn leads to the secretion of IL-6 and TNF. In addition, different miRNA formulations can be influenced by macrophages and monocytes (Squadrito et al., 2013). Yet another side effect observed include inhibition of coagulation, hepatotoxicity and activation of the complement cascade. Therefore, off target effects limit the therapeutic applicability. Current techniques to improve the delivering ncRNA-delivering include their chemical modifications, packaging into liposomes, viral delivering and their loading on nanoparticles (summarized e.g., in Sehgal et al., 2013; Chery, 2016; Grijalvo et al., 2018).

## Future Perspectives

The importance of ncRNA as therapeutic agents is increasing with remarkable progress and represents a milestone for novel therapeutic approaches. In view of the large number of reported preclinical studies on miRNA, only a few have entered clinical trials. The promising field of lncRNA is new and further experimental work is needed to understand their potential as therapeutic targets. A clear understanding on the different miRNA mechanisms such as decoy activity and 5’UTR regulatory activity is required to understand the cellular circuits and network-associated miRNA. The efficiency of the miRNA delivery system must be ensured by improving the chemical design of miRNA mimics or antagomirs. Although the adenovirus- mediated delivery of miRNA seems favorable and effective, but the adverse immune response impedes their functional capability in therapy. A patent on novel miRNA gene fragment HAAVmiR in combination with adenovirus and multiple copies of miR-122 has been filed by the group of Xu and colleagues in an attempt to reduce or eliminate contaminating effects of viral particles. Finally, the combination of miRNA or lncRNA-targeting therapeutics along with other biological drugs could bring out the efficacy of treatment. Due to the involvement of multiple cellular pathways in liver diseases, combinatorial treatment may target several pathways simultaneously which in turn can be a viable option to modulate different aspects of liver diseases.

## Author Contributions

All authors listed have made a substantial, direct and intellectual contribution to the work, and approved it for publication.

## Conflict of Interest Statement

The authors declare that the research was conducted in the absence of any commercial or financial relationships that could be construed as a potential conflict of interest. The reviewer ZL and handling Editor declared their shared affiliation.
